# Supplementary material for: Chromothripsis during telomere crisis is independent of NHEJ, and consistent with a replicative origin
Source: Genome Res. 2019 May;29(5):737–49. doi: 10.1101/gr.240705.118 (PMC6499312; doi:10.1101/gr.240705.118)
Supplement: Supplemental Material [file supp_gr.240705.118_Supplemental_file_1.zip › contigs/annotated_contigs/DB89/contig.2.DB89_length_645_mean_cov_4.74263565891.docx]

**DB89_length_645_mean_cov_4.74263565891**

```
ATTTATTAGTTATGTTTTTCCAAAGGCAAAGAATGATGATTAGGTATTTTATAATCTCACATTCAACATGGAAATTTACTTAAATTGTA
 >chr4:126712282-126712704 + E=3e-230
TAGCATAACAATTTATGGCTTTAAAATCTTTGGCCAGGTTTGGTGGCTCACACCTGTAATCCCAGAACTTTGGGAGGCCAAGGTGGGCA

GATCACTTGAGGTCAGGAGTTTGAGATCAGACTGGCCAACATGGTGAAACTTAGGCTCTACTAAAAATACAAAAATTAGCCGAGAGTGG

TGGTGGGAGTCTGTAATCCCCAGCTACTTGGGAGGCTGAGGCAGGAGAATCACTTGAACCTGGGAGGCAGAGGCTGCAGTCAGCCGAGA

TTGTACCACTGCACTCCAGCCTGGGCAACAGAGTGAAACTCTGTCTTACAAAAAACAAAAAAAAAA|GAGAAAGAAAAAGAAAG|AGCC
                                                                                    >ch
AAACATTTGTCTTCTTTGTGAGATCCCTCCCTACAACTCCAGACTTCATTTCTTGGGCCCTCACCAATGATAATTTCTGGAGTCTACAT
r4:126700715-126700921 - E=3e-112
CTGTAGTAGGAGCAGACGTGGGCATCCACATATAGGAGGACGTGCAGTTACTAGAGTTCCTCTAGTCTTTAGTTACTGTTTTATACGTG

ATTTTTGAGATGCCTGCTACCTAG
```
